# Correction: Improved sensitivity, safety, and rapidity of COVID-19 tests by replacing viral storage solution with lysis buffer

**DOI:** 10.1371/journal.pone.0314219

**Published:** 2024-11-14

**Authors:** Oran Erster, Omer Shkedi, Gil Benedek, Eyal Zilber, Itay Varkovitzky, Rachel Shirazi, Dorit Oriya Shorka, Yuval Cohen, Tzachi Bar, Rafi Yechieli, Michal Tepperberg Oikawa, Dana Venkert, Michal Linial, Esther Oiknine-Djian, Michal Mandelboim, Zvi Livneh, Gilat Shenhav-Saltzman, Ella Mendelson, Dana Wolf, Moran Szwarcwort-Cohen, Orna Mor, Yair Lewis, Danny Zeevi

The ninth author’s name is spelled incorrectly. The correct name is: Tzachi Bar.
